# Thromboangiitis obliterans plasma-derived exosomal miR-223-5p inhibits cell viability and promotes cell apoptosis of human vascular smooth muscle cells by targeting VCAM1

**DOI:** 10.1080/07853890.2021.1949487

**Published:** 2021-07-14

**Authors:** Ying Deng, Jindong Tong, Weijun Shi, Zhongyi Tian, Bo Yu, Jingdong Tang

**Affiliations:** Department of Vascular Surgery, Shanghai Pudong Hospital Affiliated to Fudan University, Shanghai, China

**Keywords:** Circulating exosomal miRNAs, exosomes, cell apoptosis, vascular smooth muscle cells, bioinformatics analysis

## Abstract

**Background:** Exosomes-encapsulated microRNAs (miRNAs) have been established to be implicated in the pathogenesis of different diseases. Nevertheless, circulating exosomal miRNAs of thromboangiitis obliterans (TAO) remains poorly understood. This study aimed to explore the effects of exosomal miRNAs associated with TAO on human vascular smooth muscle cells (HVSMCs).

**Methods:** The exosomes were isolated from the plasma of TAO patients and normal controls and then were sent for small RNA sequencing. Differentially expressed miRNAs (DE-miRNAs) were identified by bioinformatics analysis and were confirmed by RT-qPCR. After that, PKH67 staining was used to label exosomes and co-cultured with HVSMCs. Cell viability and apoptosis were, respectively, tested by CCK-8 assay and flow cytometry. Finally, dual-luciferase reporter assay was used to confirm the downstream targets of miR-223-5p.

**Results:** A total of 39 DE-miRNAs were identified between TAO patients and normal controls, of which, miR-223-5p was one of the most significantly up-regulated miRNAs. TAO plasma-derived exosomes or miR-223-5p mimics inhibited cell viability of HVSMCs and promoted cell apoptosis. The pro-apoptotic effect of TAO plasma-derived exosomes was alleviated by miR-223-5p inhibitor. Additionally, the expressions of VCAM1 and IGF1R were down-regulated by exosomes and miR-223-5p mimics, and were abrogated by miR-223-5p inhibitor. Dual-luciferase report showed that VCAM1 was the target of miR-223-5p.

**Conclusions:** Our findings imply that circulating exosomal miR-223-5p may play an essential role in the pathogenesis of TAO, and provide a basis for miR-6515-5p/VCAM1 as novel therapeutic targets and pathways for TAO treatment.

## Introduction

Thromboangiitis obliterans (TAO), also called Buerger’s disease, is a non-atherosclerotic, inflammatory vascular disorder that primarily affects small- or medium-sized arteries and veins in extremities [1]. TAO is closely associated with tobacco exposure and is characterized by the presence of a highly inflammatory thrombus in the affected vessels [2]. The vascular event-free survival of TAO patients is reported to be 41% at 5 years and 24% at 10 years [3]. Although more than a century has passed since its discovery, the pathogenesis of TAO remains elusive [4].

Exosomes are the smallest membrane vesicles that carry a load of functional proteins, mRNAs and microRNAs (miRNAs), acting as key messengers for material and information communication among cells [5]. Accumulating evidences suggest that exosomes can enhance angiogenesis, suppress ventricular remodelling and play important regulatory roles in inflammation and hold therapeutic potential for inflammatory diseases [6–8]. Circulating exosomal miRNAs have been demonstrated to be promising biomarkers of various diseases, such as Parkinson’s disease [9] and several types of cancers [10,11]. More importantly, a recent report shows that exosomes may be involved in the pathogenesis of vasculitis through regulating inflammation, autoimmunity, pro-coagulation and endothelial damage/dysfunction [12]. Nevertheless, few studies discuss the biological functions of circulating exosomal miRNAs in the pathogenesis of TAO.

In the present study, we investigated the miRNA expression profiles of the exosomes isolated from plasma of TAO patients and paired normal control subjects. Differentially expressed miRNAs (DE-miRNAs) were identified between the exosomes isolated from the plasma of TAO patients and control subjects and then were submitted for functional analyses. Since miR-223-5p was verified to be significantly higher in the TAO plasma-derived exosomes than that in the control exosomes by real-time quantitative PCR (RT-qPCR), miR-223-5p was chosen for further experiments. After that, the effects of miR-223-5p and TAO plasma-derived exosomes on human vascular smooth muscle cells (HVSMCs) were investigated, and their related mechanisms were explored. Our study is beneficial for understanding the possible molecular mechanisms underlying the role of TAO plasma-derived exosomes in TAO development.

## Materials and methods

### Cell culture and treatment

HVSMCs and HEK-T293 cells were obtained from the Cell Bank of Wuhan University (Wuhan, China). HVSMCs were cultured in F12K medium (Gibco, Invitrogen, Carlsbad, CA, USA) with 10% foetal bovine serum (FBS, Gibco, Invitrogen, USA) and 1% penicillin/streptomycin (Gibco, Invitrogen, USA); while HEK-T293 cells were cultured in Dulbecco’s modified Eagle’s medium (DMEM, Gibco, Invitrogen, USA) supplemented with 10% FBS and 1% penicillin/streptomycin.

### Plasma sample collection

We used blood samples from 3 patients who were diagnosed with TAO in Shanghai Pudong Hospital Affiliated to Fudan University and 3 healthy controls. The blood samples (10 mL) were taken from the donators using blood taking needles and EDTA anticoagulant tubes and then were centrifuged at 4 °C at 1900× *g* for 10 min. After that, the suspension was transferred to a new tube and centrifuged at 4 °C at 3000× *g* for 15 min. Subsequently, the suspension was transferred to a new tube and stored at −80 °C for further exosomes isolation. This study was approved by the ethical committee of Shanghai Pudong Hospital Affiliated to Fudan University and written informed consent was received from each participant.

### Exosomes isolation and identification

Exosomes in the obtained plasma samples were isolated and purified by ultracentrifugation according to the standard protocol as described previously [13]. Briefly, the plasma was centrifuged at 12,000× *g* for 30 min to remove cells. Then, the supernatant was filtered on a 0.22-µm pore membrane to remove large membrane vesicles. Thereafter, the supernatant was ultracentrifuged at 120,000× *g* for 60 min at 4 °C. The resulting pellets were resuspendedwith10 ml PBS and then ultracentrifuged at 120,000× *g* for another 60 min at 4 °C.

Exosomes size distribution was assessed by nanoparticle tracking analysis (NTA) using the NanoSight LM10-HS system (NS300, NanoSight, Amesbury, UK) according to the manufacturer’s instructions. After that, the morphology of purified exosomes was observed under a transmission electron microscope (TEM, JEOL, USA Inc., Peabody, MA, USA) at 120 kV [14]. Briefly, exosome suspensions (5 µL) were applied to copper mesh formvar-coated carbon stabilized grids. Ten minutes later, the remnant suspensions were wicked off by using filter paper. Subsequently, 2% phosphotungstic acid (10 µL, pH = 6.5) was added to the grids prior to 2-min staining at room temperature. Grids were then allowed to dry before observation. Finally, western blot was used to determine the expression of CD63, CD9 and 81, as well as the expression of negative controls (NC, calnexin and albumin).

### Western blot analysis

Total protein was isolated from the obtained exosomes using RIPA protein lysis buffer (Beyotime Biotechnology, Shanghai, China), and the protein concentrations were measured using a BCA protein assay kit (Wuhan Boster Biological Technology, Ltd.) based on the manufacturer’s protocol. After that, protein samples (20 µg) were separated *via* 10% SDS-PAGE, transferred to PVDF membranes and blocked with 5% skimmed milk for 2 h at 37 °C. Thereafter, the membranes were, respectively, incubated with anti-CD63 antibody (1:1000, No. A5271, ABclonal), anti-CD81 antibody (1:1000, No. ab109201, Abcam), anti-CD9 antibody (1:1000, No. ab92726, Abcam), anti-calnexin antibody (1:1000, No. 10427-2-AP, Proteintech) and anti-albumin antibody (1:1000, No. 16475-1-AP, Proteintech) overnight. After washing with PBST (0.05% Tween 20 in PBS) three times, the membranes were incubated with horseradish peroxidase-conjugated goat anti-rabbit IgG (1:3000, No. 111-035-003, Jackson ImmunoResearch) at 37 °C for 2 h. Following washing three times with PBST, protein bands were visualized using the Millipore electrochemiluminescence ECL system.

### MiRNA library construction and sequencing

Total RNA was extracted from exosomes using RNAiso Plus (Takara, China) according to the standard method. MiRNA library construction and sequencing were done by Yanzai Biotechnology (Shanghai) Co. Ltd. (Shanghai, China). Small RNAs between 18 and 30 nucleotides were used for library preparation. Small RNAs were amplified by PCR and sequenced. The obtained clean reads were aligned with Rfam [15] database (version 10.0) using blast software with the threshold cut-off of E-value ≤0.01. The un-annotated small RNAs were used to predict novel miRNAs using miRDeep 2 software [16].

### Screening of DE-miRNAs and functional analyses

DE-miRNAs were screened between the exosomes from the TAO and normal control samples with the criteria of |log_2_(fold change)| ≥ 1.5 and FDR < 0.05 by using DEseq (1.18.0) package of R language [17]. Target genes of the identified DE-miRNAs were predicted based on miRanda database (http://www.microrna.org/microrna/home.do). Afterwards, gene ontology (GO) [18] function and Kyoto Encyclopedia of Genes and Genomes (KEGG) [19] pathway enrichment analyses were further performed using super geometric distribution test statistics.

### Quantification of exosomal miRNA by RT-qPCR

Total RNA (0.5 µg) was reversed transcribed to cDNA with specific RT-Primer by using PrimeScript™ RT Master Mix Kit (Takara, Dalian, China) for examining miRNA levels or using PrimeScript™ II 1st Strand cDNA Synthesis Kit (Takara, Dalian, China) for examining mRNA expressions. The cDNA products were then amplified using the Power SYBR Green PCR Master Mix kit (Thermo Fisher Scientific, Waltham, MA, USA). RT-qPCR reaction conditions were shown as follows: pre-denaturation at 95 °C for 2 min; 40 cycles at 95 °C for 15 s and 60 °C for 60 s; melt curve at 95 °C for 15 s, 60 °C for 60 s and 95 °C for 15 s. The sequences of all primers were displayed in Table 1. U6 was used as an internal reference, and cel-miRR-39-3p standard RNA (RiboBio Co., Ltd., Guangzhou, China) was served as an external reference. For mRNA, glyceraldehyde-3-phosphate dehydrogenase (GAPDH) was served as a housekeeping gene. The relative levels of miRNAs and the relative mRNA expressions were calculated using the 2^−ΔΔCt^ method

**Table 1.** The primer sequences of all primers for qRT-PCR.

Primer	Sequence
VCAM1-hF	GGGAAGATGGTCGTGATCCTT
VCAM1-hR	TCTGGGGTGGTCTCGATTTTA
IGF1R-hF	TCGACATCCGCAACGACTATC
IGF1R-hR	CCAGGGCGTAGTTGTAGAAGAG
GAPDH-hF	TGACAACTTTGGTATCGTGGAAGG
GAPDH-hR	AGGCAGGGATGATGTTCTGGAGAG
hsa-miR-let-7b-3p-RT	GTCGTATCCAGTGCAGGGTCCGAGGTATTCGCACTGGATACGACGGGAAG
hsa-miR-let-7b-3p-F	GCCTATACAACCTACTGC
hsa-miR-127-3p-RT	GTCGTATCCAGTGCAGGGTCCGAGGTATTCGCACTGGATACGACAGCCAA
hsa-miR-127-3p-F	GCTCGGATCCGTCTGAG
hsa-miR-223-5p-RT	GTCGTATCCAGTGCAGGGTCCGAGGTATTCGCACTGGATACGACAACTCA
hsa-miR-223-5p-F	GCCGTGTATTTGACAAGC
novel19_mature-RT	GTCGTATCCAGTGCAGGGTCCGAGGTATTCGCACTGGATACGACCCCACA
novel19_mature-F	GCGAATCAGTGAGAC
novel227_mature-RT	GTCGTATCCAGTGCAGGGTCCGAGGTATTCGCACTGGATACGACCCTTCC
novel227_mature-F	GCGGGAAACTCTGGT
hsa-miR-6529-5p-RT	GTCGTATCCAGTGCAGGGTCCGAGGTATTCGCACTGGATACGACCACTCT
hsa-miR-6529-5p-F	GCGAGAGATCAGAGGCGC
Universal downstream	GTGCAGGGTCCGAGGT
hsa-U6-RT	GTCGTATCCAGTGCAGGGTCCGAGGTATTCGCACTGGATACGACAAAATATG
hsa-U6-F	CTCGCTTCGGCAGCACA
hsa-U6-R	AACGCTTCACGAATTTGCGT
cel-miR-39-RT	GTCGTATCCAGTGCAGGGTCCGAGGTATTCGCACTGGATACGACCAAGCT
cel-miR-39-F	GGCCTCACCGGGTGTAAATCAG

### Confocal scanning microscopy analysis of exosomes internalization

Exosomes internalization in HVSMCs was explored by labelling with PKH67 (green fluorescent) using a commercial kit (PKH67GL-1KT; Sigma-Aldrich, USA) as previously described [20]. Briefly, 700 µL of exosomes (about 6.608 × 10^10^ particles) isolated from plasma of TAO patients were added to Diluent C (1300 µL), and then 16 µL PKH67 dye with 2 mL Diluent C were added. The mixture was incubated at room temperature for 5 min, and 1% bovine serum albumin (4 mL BSA; Sigma-Aldrich, USA) was added to bind the excess dye. Subsequently, the mixture was centrifuged at 4 °C at 120,000× *g* for 90 min, and the sediment (PKH67-labeled exosomes) was resuspended with 300 µL PBS for use.

HVSMCs were seeded into a 24-well plate at density of 1 × 10^5^ cells/well and cultured overnight. On the next day, 10 µg/mL PKH67-labeled exosomes were added to the cells. After co-incubated for 24 h and 48 h, the cells were fixed with 4% paraformaldehyde for20 min and then were treated with 0.1% Triton X-100 at room temperature for 20 min. After washing, the cells were stained with DAPI (4,6-diamidino-2-phenylindole, Sigma, Dalian, China) and were observed under a laser confocal scanning microscope (Leica, Wetzlar and Mannheim, Germany).

### Cell grouping

MiR-223-5p mimics, NC mimics and miR-223-5p inhibitors were purchased from Ribobio (Guangzhou, China). HVSMCs were seed into a 24-well plate at density of 1 × 10^5^ cells/well and cultured overnight. On the next day, the cells were divided into five groups: blank group, NC group, Exos group, miRNA mimics group and miRNA inhibitor + Exos group. The cells in the NC group and miRNA mimics group were, respectively, transfected with NC mimics and miR-223-5p mimics using Lipofectamine 2000 (Invitrogen, Carlsbad, USA) according to the manufacturer’s instructions. The cells in the Exos group were treated with 100 µg TAO plasma-derived exosomes. The cells in the miRNA inhibitor + Exos group were firstly transfected with miR-223-5p inhibitor using Lipofectamine 2000 and then were treated with 100 µg TAO plasma-derived exosomes. The cells in the blank group were without treatments.

### Cell viability and apoptosis

Cell viability of HVSMCs with different treatments was detected using Cell Counting Kit-8 (CCK-8, Beyotime Institute of Biotechnology, Nanjing, China) according to the manufacturer’s instructions. Briefly, the cells with different treatments were cultured for 24 h, 48 h and 72 h, and then 10 µL of CCK-8 reagent was added to each well. After 2 h of incubation, the absorbance was measured at 450 nm using a microplate reader (Multiskan MK3; Thermo Fisher Scientific).

After that, cell apoptosis of HVSMCs with different treatments was determined using Annexin V-FITC apoptosis detection kit (BD Pharmingen, CA, USA) following the manufacturer’s protocols. Briefly, the cells with different treatments were harvested and centrifuged at 1000 rpm for 5 min. After washing with PBS, the cells were resuspended with 1× Binding Buffer (100 µL). Thereafter, 5 µL FITC-Annexin V and 5 µL PI (50 µg/mL) were added and incubated at 25 °C in the dark for 15 min. After adding 400 µL 1× Binding Buffer, the images were acquired by a BD FACScalibur flow cytometer (CA, USA), and cell apoptosis rates were analysed. All experiments were repeated in triple.

### Dual-luciferase reporter assay

The sequences of hsa-miR-223-5p and VCAM-13′-untranslated region (3′-UTR) were synthesized by Ribobio (Guangzhou, China). The pGL3-basic vector (General Biosystems, Chuzhou, China) was used to construct the 3′-UTR vascular endothelial cell adhesion molecule 1 (VCAM1) report plasmid (pGL3-VCAM1). After that, pGL3-VCAM1 (500 ng) or pGL3-basic vector (500 ng) was co-transfected to HEK-T293 cells with miR-223-5p mimics (100 nM) or NC mimics (100 nM) using Lipofectamine 2000. At 48 h post-transfection, dual-luciferase reporter assay system (Promega, Madison, USA) was employed to measure the luciferase activity according to the standard protocol.

### Statistical analysis

All statistical analysis was performed using GraphPad Prism 5 software (GraphPad, San Diego, USA). Student’s *t*-test was used to compare two groups. For more than two groups’ comparison, analysis of variance (ANOVA) was used. *p* < 0.05 suggests significant difference.

## Results

### Identification of exosomes from the plasma of TAO patients and control subjects

**Figure 1.**
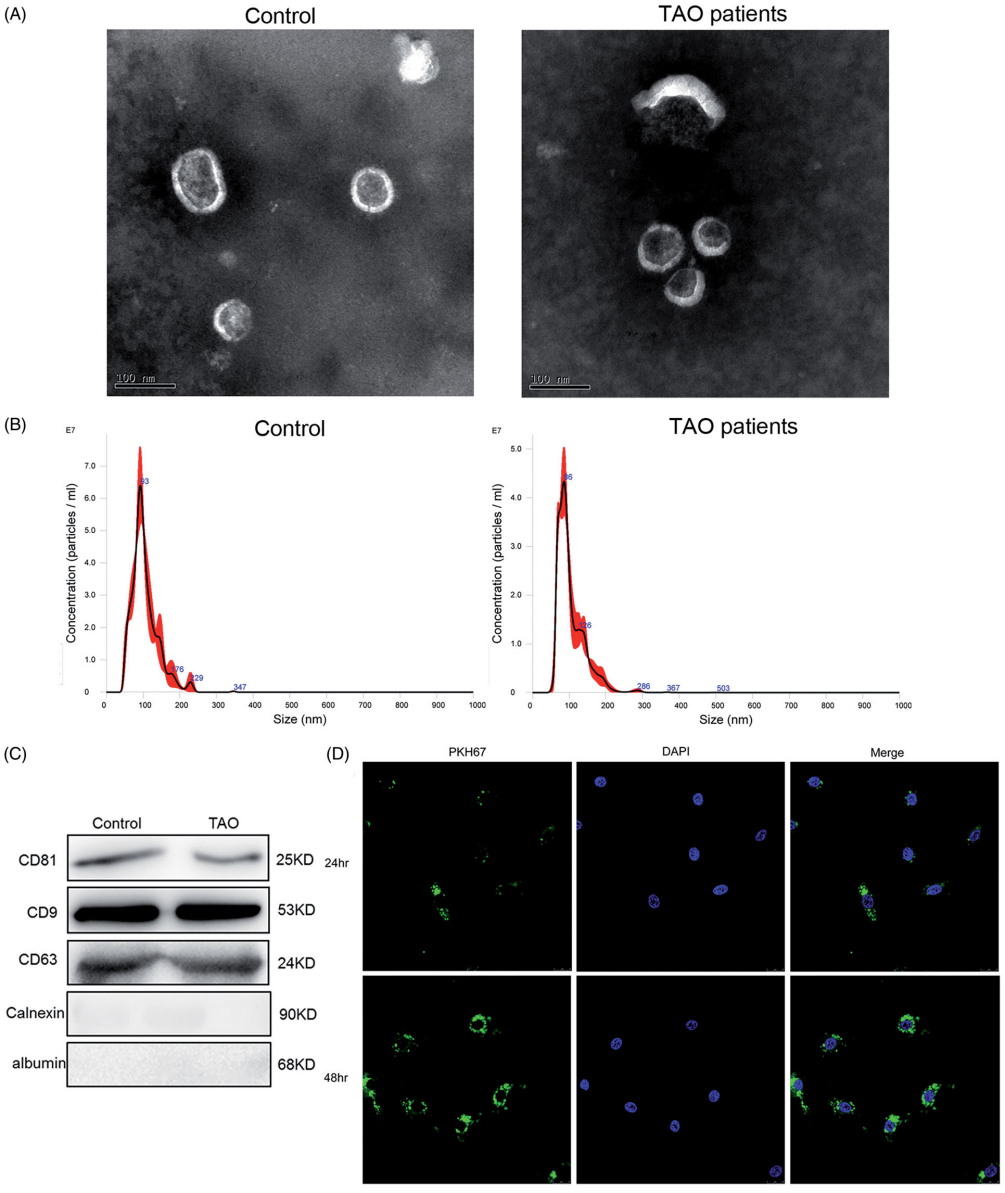
Characterization of plasma-derived exosomes from thromboangiitis obliterans (TAO) patients and control subjects. (A) Representative transmission electron microscopic images of exosomes (scale bar, 100 nm). (B) NTA analysis of the plasma-derived exosomes from TAO patients and control subjects. (C) Expression of CD81, CD9, CD63, calnexin and albumin determined using western blot. (D) PKH67-labelled exosomes could be taken up by human vascular smooth muscle cells (HVSMCs) after co-cultured for 24 h and 48 h (magnification, 400×).

Exosomes were isolated from the plasma samples of three TAO patients and three paired control subjects, respectively. Demographic data of these patients and normal subjects were not significantly different. TEM results showed that the purified exosomes were typical particles that were consistently 40–100 nm in diameter (Figure 1(A)). NTA showed that the plasma-derived exosomes extracted from TAO patients and control subjects had a similar distribution of particles size (Figure 1(B)); as well as the concentrations of exosomes isolated from the plasma of TAO patients and healthy controls were 9.44 × 101° particles/mL and 1.456 × 10^10^ particles/mL, respectively. Thereafter, CD81, CD9 and CD63 are established as markers of exosomes, and calnexin and albumin are served as NCs [21]. We therefore detected their expressions using western blot, and found that CD81, CD9 and CD63 were all expressed in the exosomes, and calnexin and albumin were not expressed (Figure 1(C)). These results indicated that exosomes were successfully isolated from the plasma of TAO patients and control subjects.

In addition, to examine whether TAO plasma-derived exosomes can be taken up and internalized by HVSMCs, PKH67was used to label exosomes (green fluorescence). After co-cultured with HVSMCs for 24 h and 48 h, most HVSMCs displayed intracellular green fluorescence, as well as more exosomes, were internalized by HVSMCs at 48 h than that at 24 h (Figure 1(D)). These results suggested that TAO plasma-derived exosomes could be taken up and internalized by HVSMCs.

### MiRNA expression profiling of exosomes and screening for DE-miRNAs

**Figure 2.**
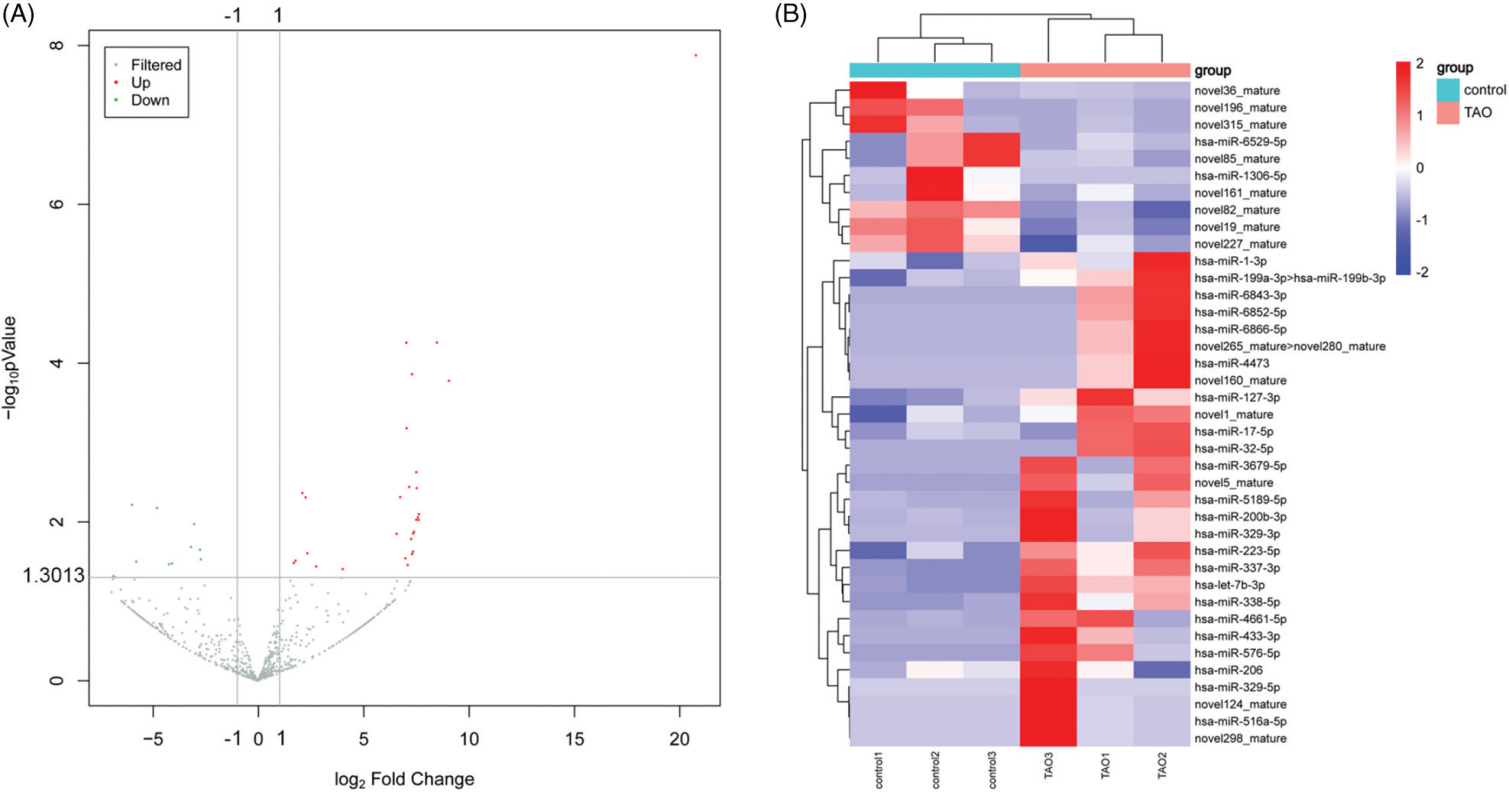
Screening of differentially expressed microRNAs (DE-miRNAs) between TAO patients and control subjects. (A) The volcano plot of DE-miRNAs. The dots above y = 1.3013 (−log0.05) and to the right of x = 1 represented the up-regulation; and the dots above y = 1.3013 (−log0.05) and to the left of x = −1 represented the down-regulation. (B) The heatmap of DE-miRNAs.

In order to identify the promising miRNAs in exosomes that may be involved in the pathogenesis of TAO, miRNA-seq was used to analyse the miRNAs profiles of the exosomes derived from TAO patients and control subjects. It was found that the produced clean reads were varied from 12.08 M to 16.03 M in all samples, and the percentage of the annotated miRNAs in total RNAs ranged from 8.88% to 24.69%. After analysing, a total of 523 known miRNAs and 309novel predicted miRNAs were obtained (Supplementary Table S1). Based on the criteria of |log_2_(fold change)| ≥ 1.5 and FDR < 0.05, 39 DE-miRNAs were identified in the comparison of the TAO patients versus the control individuals, including 10 down-regulated and 29 up-regulated miRNAs (Figure 2(A), Supplementary Table S2). Afterwards, the heatmap distribution of these DE-miRNAs expressions was shown in Figure 2(B).

### GO function and KEGG pathway enrichment analyses of the DE-miRNAs

**Figure 3.**
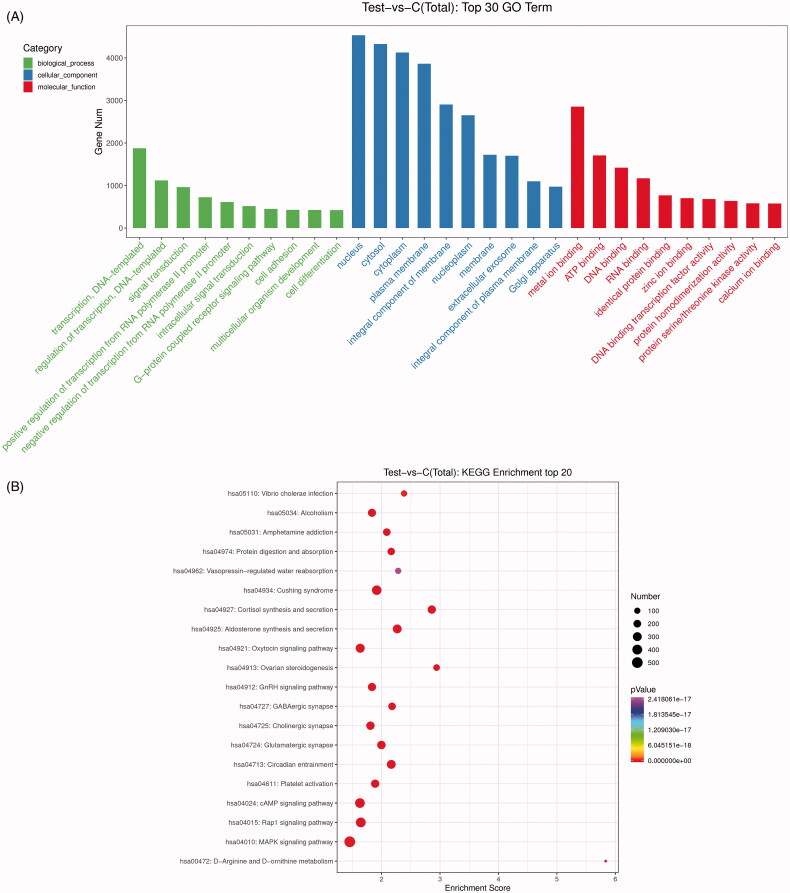
Gene ontology (GO) function and Kyoto Encyclopaedia of Genes and Genomes (KEGG) pathway enrichment analyses for the identified DE-miRNAs. (A) The top 30 significant GO terms (biological process, cellular component and molecular function). (B) The top 20 significant KEGG pathways. Number: the count of genes significantly enriched in a pathway.

To decipher the possible functions of the identified DE-miRNAs in TAO, target genes were predicted using miRanda database. A total of 19,709 predicted target genes were acquired and then submitted for GO and KEGG pathway enrichment analyses. GO terms results showed that the identified DE-miRNAs were significantly related to regulation of transcription and signal transduction in biological process; and nucleus, cytosol and cytoplasm in cellular component; as well as metal ion binding, ATP binding and DNA binding in molecular function (Figure 3(A)). Additionally, the top 20 KEGG pathways were found with *p* < 0.05 (Figure 3(B)). It was shown that these DE-miRNAs were significantly enriched in MAPK signalling pathway, Rap1 signalling pathway, cAMP signalling pathway, oxytocin signalling pathway and GnRH signalling pathway.

### Verification of exosomal DE-miRNAs using RT-qPCR

**Figure 4.**
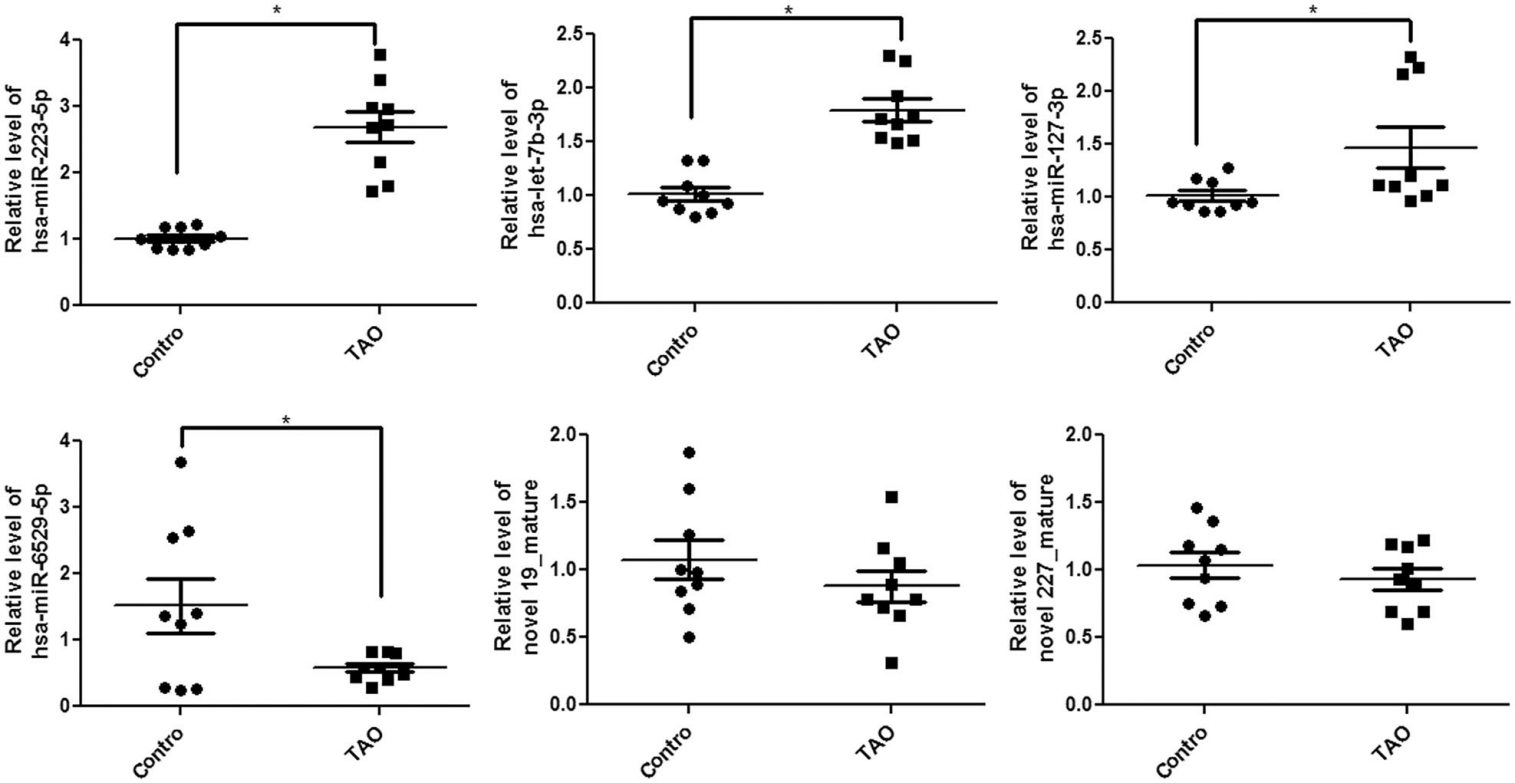
Circulating exosomal expression levels of miR-223-5p, let-7b-3p, miR-6529-5p, miR-127-3p, novel 19_mature and novel 227_mature in the exosomes isolated from the plasma of TAO patients and control subjects by real-time quantitative PCR. Experiments are performed in triplicated. *p < 0.05 vs. control subjects.

After that, the top 6 DE-miRNAs, including three up-regulated (miR-223-5p, miR-let-7b-3p and miR-127-3p) and three down-regulated (miR-6529-5p, novel 19_mature and novel 227_mature) miRNAs, were selected for experimental validation by RT-qPCR. Compared with the control exosomes, the levels of miR-223-5p, miR-let-7b-3p and miR-127-3p were significantly increased in the exosomes isolated from the plasma of TAO patients (*p* < 0.05); whereas the level of miR-6529-5p was remarkably decreased in the exosomes isolated from the plasma of TAO patients (*p* < 0.05, Figure 4). However, no significant differences in novel 19_mature and novel 227_mature levels were found among the exosomes isolated from the plasma of TAO patients and control individuals (*p* > 0.05, Figure 4). The results showed that the consistency rate between sequencing analyses and RT-qPCR results was 66.67%, which indicated a relatively high reliability of the sequencing results. Furthermore, since miR-223-5p had a more obvious elevation than miR-let-7b-3p and miR-127-3p, we focussed on miR-223-5p in the following experiments.

### TAO-derived exosomes suppressed cell viability and promoted cell apoptosis in HVSMCs through up-regulating miR-223-5p

**Figure 5.**
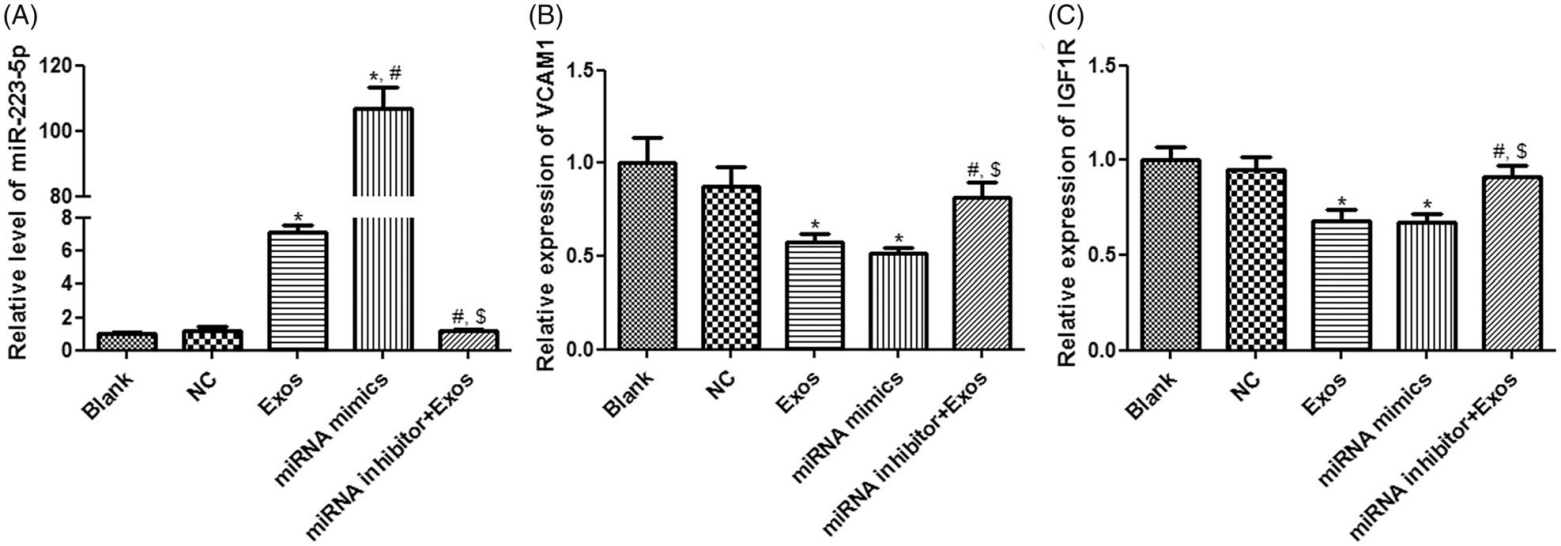
Effects of miR-223-5p and exosomes on HVSMCs with different treatments. (A) The level ofmiR-223-5pindifferent groups. (B) The expression of VCAM1 in different groups. (C) The expression of IGF1R in different groups. *p < 0.05 vs. blank group; ^#^*p* < 0.05 vs. Exos group; ^$^*p* < 0.05 vs. miRNA mimics group.

In order to understand whether TAO plasma-derived exosomes and miR-223-5p could affect cell viability and apoptosis of HVSMCs, miR-223-5p mimics and miR-223-5p inhibitor were, respectively, used to overexpress and knockdown miR-223-5p. It was found that there was no significant difference in miR-223-5p level between blank and NC groups (*p* > 0.05, Figure 5(A)). After the cells were incubated with TAO plasma-derived exosomes and miR-223-5p mimics, the level of miR-223-5p was significantly increased (*p* < 0.05), and its level in the miRNA mimics group was increased by more than 100 folds compared with the control group (Figure 5(A)). However, miR-223-5p inhibitor combined with TAO plasma-derived exosomes restored miR-223-5p levels induced by exosomes to a similar level in the blank group (Figure 5(A)). After that, *VCAM1* and insulin-like growth factor-1 receptor (IGF1R) expressions were determined. No significant differences were found in the *VCAM1* and *IGF1R* expressions between blank and NC groups (*p* > 0.05, Figure 5(B,C)). Compared with the blank group, *VCAM1* and *IGF1R* expressions were significantly down-regulated in the Exos and miRNA mimics groups (*p* < 0.05, Figure 5(B,C)); whereas their expressions in the miRNA inhibitor + Exos group were evidently higher than those in the Exos and miRNA mimics groups (*p* < 0.05, Figure 5(B,C)).

**Figure 6.**
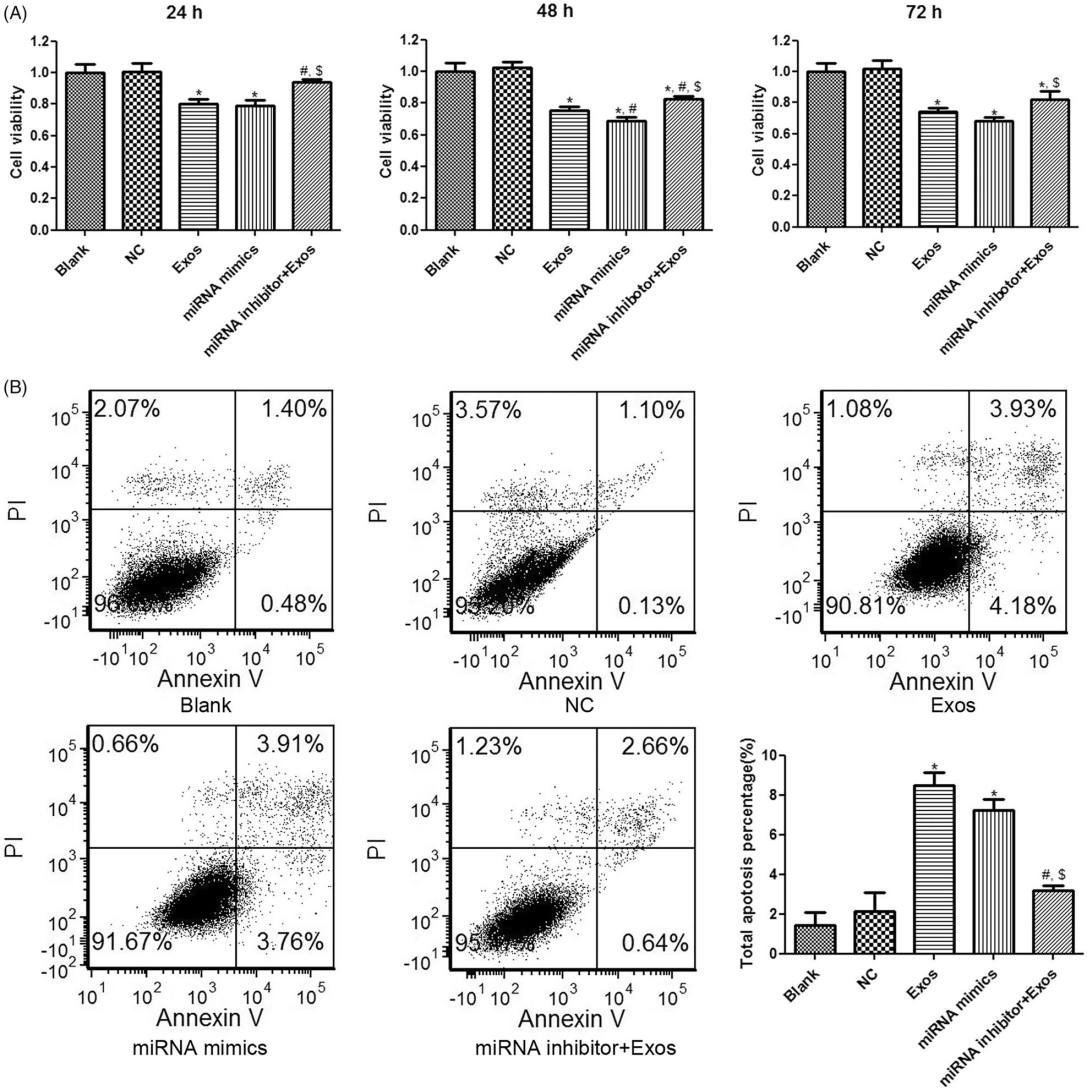
Effects of miR-223-5p and exosomes on cell viability and apoptosis of HVSMCs with different treatments. (A) Cell viability of HVSMCs with different treatments examined by CCK-8 assay. (B) Cell apoptosis of HVSMCs with different treatments measured by flow cytometry. *p < 0.05 vs. blank group; ^#^*p* < 0.05 vs. Exos group; ^$^*p* < 0.05 vs. miRNA mimics group.

Thereafter, cell viability and apoptosis of HVSMCs in response to different treatments was evaluated. Both TAO plasma-isolated exosomes and miR-223-5p mimics transfection significantly suppressed cell viability of HVSMCs when compared to the control group at 24 h, 48 h and 72 h (*p* < 0.05, Figure 6(A)). Combined treatment with exosomes and miR-223-5p inhibitor significantly restored the cell viability compared to the treatment with exosomes alone at all three time points (*p* < 0.05, Figure 6(A)), suggesting that cell viability suppression of HVSMCs by TAO exosomes was mediated by miR-223-5p overexpression. In addition, flow cytometry results showed that the cell apoptosis was significantly increased in the Exos and miRNA mimics groups relative to the blank group (*p* < 0.05, Figure 6(B)). The miR-223-5p down-regulation by miR-223-5p inhibitor significantly ameliorated cell apoptosis induced by TAO circulating exosomes (*p* < 0.05, Figure 6(B)). These results implied that TAO circulating exosomes-induced cell apoptosis was mediated by up-regulating miR-223-5p in HVSMCs.

### VCAM1directly binds with miR-223-5p

**Figure 7.**
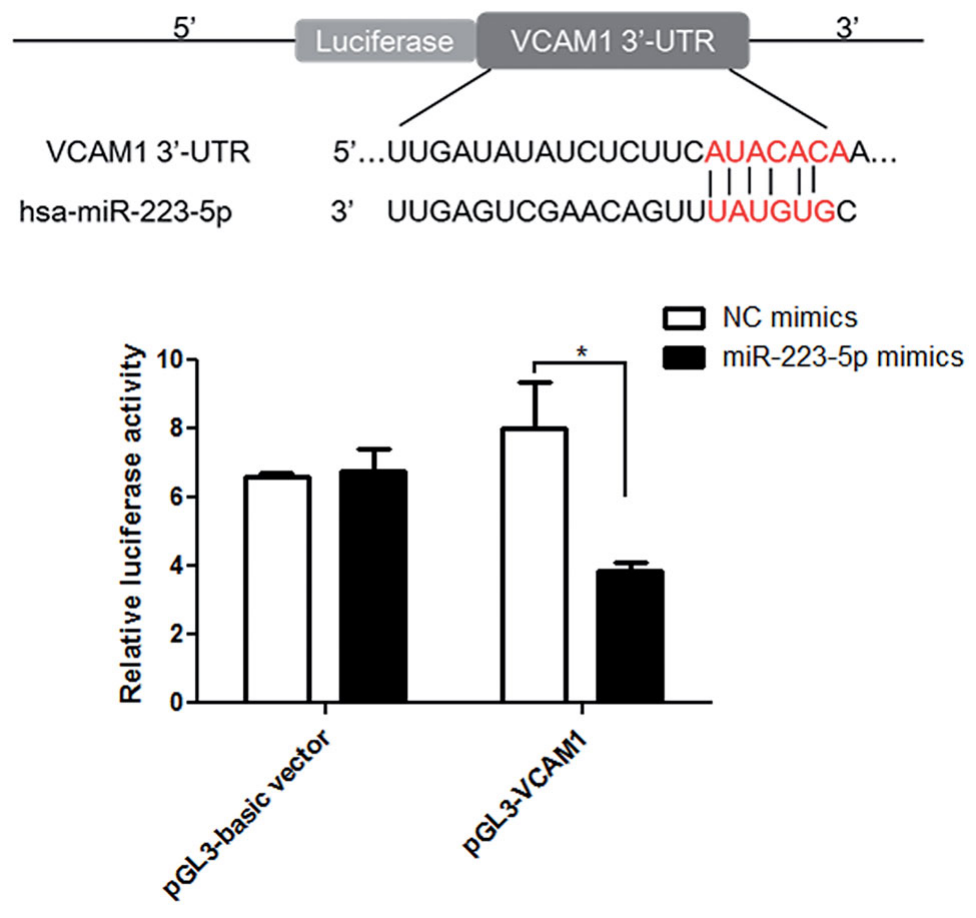
Interaction between miR-223-5p and VCAM-1 determined using luciferase reporter assay.*p < 0.05 vs. NC mimics.

TargetScan Human 7.1 was used to predict that VCAM1 was the downstream gene of miR-223-5p (Figure 7), because the 3′-UTR of VCAM1 contained the binding sites of miR-223-5p. Further to confirm the conclusion, dual-luciferase reporter assay was used. It was found that there was no significant difference in the relative luciferase activity among the pGL3-basic vector transfected with NC mimics, pGL-basic vector transfected with miR-223-5p mimics and pGL-VCAM1 transfected with NC mimics (*p* > 0.05, Figure 7). However, in the pGL-VCAM1, the relative luciferase activity after transfected with miR-223-5p was significantly decreased compared to that transfected with NC mimics (*p* < 0.05, Figure 7). These results indicated that VCAM-1 was a downstream target of miR-223-5p.

## Discussion

Exosomal miRNAs play critical roles in the progression of many diseases and have been increasingly recognized as potential non-invasive biomarkers for clinical applications [22]. Although clinical significance of circulating exosomal miRNAs for several types of human cancers has been demonstrated previously [23], more studies are required to elucidate the involvement of exosomal miRNAs in the development of TAO. To the best of our knowledge, our study is the first study to focus on the miRNAs profiles of circulating exosomes from TAO patients. We purified exosomes from the plasma of TAO patients and normal control subjects and compared the exosomal miRNAs expression profiles. It was found that 39 DE-miRNAs were identified, and were enriched in a range of regulation of transcription-related biological processes, and various signalling pathways such as cAMP signalling pathway and MAPK pathway, which are in agreement with previous studies showing that the two pathways play a role in inflammatory diseases and may serve as therapeutic targets [24, 25]. Moreover, RT-qPCR further verified the sequencing analyses and indicated a relatively high reliability of the sequencing results.

Exosomes, as the carrier of proteins and nucleic acids, can deliver the bioactive substance from their parent cells to recipient cells, thus affecting the biological functions of recipient cells [26]. In our study, we observed that TAO plasma-derived exosomes could be taken up and internalized by HVSMCs. When HUVECs were treated with TAO plasma-derived exosomes, the cell viability of HUVECs was suppressed and their apoptosis was induced. Moreover, TAO plasma-derived exosomes could significantly increase miR-223-5p level and down-regulated VCAM1 and IGF1R compared with the blank control cells. Ham et al. [27] indicated that breast cancer-derived exosomes could induce IL-6 secretion and a pro-survival phenotype in macrophages partially through gp130/STAT3 signalling. Another study found that hypoxic gastric cancer-derived could promote the proliferation, invasion, migration and epithelial-mesenchymal transition of gastric cancer cells, thus contributing to the progression and metastasis of gastric cancer. Combined with our results, it can be inferred that TAO plasma-derived exosomes can regulate viability and apoptosis of HVSMCs, thereby promoting the occurrence and development of TAO.

Since miR-223-5p showed a more obvious up-regulation in our experiments, we further explored the effects of exosomal miR-223-5p on HVSMCs. It was found that miR-223-5p overexpression significantly inhibited cell viability and induced cell apoptosis, whereas miR-223-5p down-regulation reversed TAO circulating exosomes-induced cell apoptosis and decreased cell viability in HVSMCs. These suggested that miR-223-5p mediated the pro-apoptotic effect of TAO circulating exosomes on HVSMCs. miR-223 is an important anti-inflammatory miRNA predominately present in myeloid cells and participates in post-transcriptionally regulating a cluster of genes critical for inflammation, cell proliferation and invasion [28]. The miR-223 is enriched in VSMCs and its role in VSMCs proliferation, migration and vascular remodelling has been well defined [29]. MiR-223 is also a crucial miRNA in exosome, participating in the regulation of biological functions of the recipient cells by intercellular transfer of exosomes [30]. MiR-223 duplex is comprised of double strands: the passenger strand (miR-223-5p) and the guide strand (miR-223-3p). There is *in vivo* evidence that miR-223-5p is implicated in sepsis-induced inflammation and myocardial dysfunction in mouse model [31]. A recent study reports that circulating miR-223-5p may be a diagnostic biomarker of hypertension [32]. Moreover, a growing body of evidence shows that circulating miR-223-5p or miR-223 level is associated with cigarette smoke, a critical causative factor of TAO [33, 34]. However, there is a lack of reports with regard to circulating exosomal miR-223-5p of TAO patients. Our study revealed that circulating exosomal miR-223-5p of TAO inhibited cell viability and induced cell apoptosis in HVSMCs. Circulating exosomalmiR-223-5p may play a regulatory role in the development of TAO.

In addition, VCAM-1 is an important endothelial inflammation marker involved in inflammation-related vascular adhesion and may be a promising biomarker for vascular dysfunction [35]. Increased expression of VCAM-1 is reported in thickened vascular endothelial cells and some inflammatory cells of TAO patients [36].VCAM-1 up-regulation is caused in response to stimulation of platelet-derived microparticles in human umbilical vein endothelial cells [37]. IGF-1R, a member of the insulin receptor family, participates in diverse physiological functions of the cardiac and neurological systems [38]. Decreased IGF1R expression in plaque tissue is reported to be related to clinical cardiovascular events [39]. It has been shown that platelet-secreted miR-223 advances endothelial cell apoptosis by targeting IGF1R [40]. Another study by Wang et al. found that the regulatory function of platelet miR-223 on arterial thrombosis was mediated by vascular wall IGF1R [41]. In the current article, consistently, VCAM-1 were found to be downstream targets of miR-223, as evidenced by the results that VCAM-1 and IGF-1R expression in HVSMCs were remarkably down-regulated on exposure to TAO plasma-derived exosomes, which was restored by miR-223-5p inhibitor transfection. Dual-luciferase report assay confirmed that VCAM-1 directly binds with miR-223-5p. Our results uncovered that TAO plasma-derived exosomes exerted pro-apoptotic activity on HVSMCs through miR-223-5p/VCAM-1 pathway and mediating IGF1R expression. Additionally, a recent report shows that interleukin-6 (IL-6)/signal transducer and activator of transcription (STAT) 3 pathway modulates adhesion molecules and cytoskeleton of endothelial cells in TAO by regulating the expression of VCAM-1and intercellular adhesion molecule (ICAM)-1 [42]. MiR-223 is reported to promote IL-6 production in macrophages *via* targeting STAT3 [43]. We speculate that circulating exosomal miR-223-5p may advance the development of TAO partly *via* IL-6/STAT3 pathway. However, further investigations are required to verify more speculations and provide more novel insights into the molecular mechanism of TAO.

This study inevitably has some limitations. First, the sample size was small, and further experiments with a larger sample size are essential to substantiate our findings. Second, exosomes were isolated by ultracentrifugation. Though ultracentrifugation is the most commonly used method for exosomes isolation, the amount of exosomes collected is not consistent due to small and fragile pellets or sedimentation efficiency. New method for isolation of exosomes with higher purity has been proposed in recent years [44]. Third, the exosomes were isolated from TAO patients and healthy controls. Though demographic data of these patients and normal subjects were not significantly different, the *in vivo* source of exosomes might be complicated. In addition, though we found the viability and apoptosis of HVSMCs were affected by exosomes or miR-223-5p, the molecular mechanisms and other factors are not investigated. Further research might be conducted on exosomes isolated by more advanced methods.

## Conclusion

Taken together, our findings demonstrate that plasma-derived exosomes from TAO patients suppress cell viability and induce cell apoptosis in HVSMCs. The mechanisms involved up-regulation of exosomal miR-223-5p which targets VCAM1. Our findings implied that circulating exosomal miR-223-5p may play an essential role in the pathogenesis of TAO, and provide a basis for miR-6515-5p/VCAM1 as novel therapeutic targets and pathways for TAO treatment.

## Ethical approval

This study was approved by the ethical committee of Shanghai Pudong Hospital Affiliated to Fudan University.

## Informed consent

All patients in this study provided written informed consent.

## Supplementary Material

Supplemental Material

## Data Availability

The authors confirm that the data supporting the findings of this study are available within the article.
